# Plasma BDNF is a more reliable biomarker than erythrocyte omega-3 index for the omega-3 fatty acid enrichment of brain

**DOI:** 10.1038/s41598-020-67868-9

**Published:** 2020-07-02

**Authors:** Dhavamani Sugasini, Poorna C. R. Yalagala, Papasani V. Subbaiah

**Affiliations:** 1 Division of Endocrinology and Metabolism, Department of Medicine, University of Illinois at Chicago, Chicago, IL 60612 USA; 2 Jesse Brown VA Medical Center, Chicago, IL 60612 USA

**Keywords:** Biochemistry, Neuroscience, Biomarkers, Neurology

## Abstract

Enriching brain DHA is believed to be beneficial for the prevention and treatment of several neurological diseases, including Alzheimer’s disease. An impediment in assessing the effectiveness of the treatments is the lack of a reliable biomarker for brain DHA. The commonly used erythrocyte omega-3 index is not suitable for brain because of the involvement of unique transporter at the blood brain barrier (BBB). We recently showed that dietary lysophosphatidylcholine (LPC)-DHA significantly increases brain DHA, which results in increase of brain BDNF. Since there is bidirectional transport of BDNF through the BBB, we tested the hypothesis that plasma BDNF may be used as biomarker for brain DHA enrichment. We altered the brain DHA in rats and mice over a wide range using different dietary carriers of DHA, and the correlations between the increase in brain omega-3 index with the increases in plasma BDNF and the erythrocyte index were determined. Whereas the increase in brain omega-3 index positively correlated with the increase in plasma BDNF, it negatively correlated with the erythrocyte index. These results show that the plasma BDNF is more reliable than the erythrocyte index as biomarker for assessing the effectiveness of omega-3 supplements in improving brain function.

## Introduction

The brain contains a very high concentration of the essential omega-3 fatty acid (FA) docosahexaenoic acid (DHA), which plays an important role in the normal development and function of the brain. Deficiency of DHA is associated with several neurological diseases, including Alzheimer’s, schizophrenia, Parkinson’s, and major depressive disorder^1–3^. Furthermore, epidemiologic^4^ and pre-clinical studies^5–8^ show beneficial effects of dietary omega-3 FA in the prevention and management of these diseases. Therefore, nutritional supplements such as fish oil are widely used in order to increase brain DHA with a hope to prevent these diseases or mitigate their effects. Although some beneficial effects have been reported^9–11^, majority of the controlled clinical trials using the currently available supplements failed to show improvement in Alzheimer’s disease^12–14^, Huntington’s disease^15^, or schizophrenia^16^. A possible reason for the failure of these trials is that the supplements do not significantly enrich brain DHA at clinically relevant doses, and therefore it is necessary to measure the brain DHA levels in order to test their effectiveness. Since a direct measurement of brain DHA is not possible, reliable non-invasive biomarkers are needed to determine the brain enrichment. Currently, the most widely used biomarker is the percentage of eicosapentaenoic acid (EPA) + DHA in the erythrocyte membrane lipids (omega-3 index)^17^. The basis for using this biomarker is the epidemiologic data showing that the dietary intake of omega-3 FA is positively correlated with the changes in the erythrocyte omega-3 index^18^. Furthermore, the increase in omega-3 FA of erythrocytes, following fish oil feeding correlated positively with the changes in brain DHA content in aged rats^19^, as well as neonatal baboons^20^. In contrast, other studies reported no positive correlation between erythrocyte DHA and brain DHA in swine which were fed fish oil^21^ or in weanling rats fed alpha linolenic acid^22^. The mechanism of uptake of DHA and EPA by the brain is unlike the uptake by the systemic tissues because of the involvement of a transporter at the blood brain barrier which is specific for the lysophosphatidylcholine (LPC)-form of DHA^23^, whereas most other tissues obtain their omega-3 FA via lipoprotein uptake or by exchange with plasma lipids. Therefore, the enrichment of brain DHA may not correlate with that of other tissues, including the erythrocytes. We recently demonstrated that the brain DHA can be increased by up to 100% by feeding LPC-DHA to mice and rats^24,25^. We also found an increase in the brain derived neurotrophic factor (BDNF) in the brain, concomitant with its increase in DHA levels^24^. Since DHA is known to increase the synthesis of BDNF in the brain^26,27^, and since there is a bidirectional transport of BDNF through the blood brain barrier (BBB)^28^, we investigated whether the increase in plasma BDNF can be used as a functional biomarker for the increase in brain DHA. We determined plasma BDNF levels in rats and mice whose brain DHA levels were altered over a wide range with various nutritional supplements. The results presented here show a strong positive correlation between the increase in brain DHA and the increase in plasma BDNF levels in both rats and mice. In contrast, the erythrocyte omega-3 FA levels were negatively correlated with the brain DHA levels, although they correlated positively with the increase in adipose tissue and heart. These results show that plasma BDNF level is a more reliable biomarker for the brain DHA levels compared to the erythrocyte omega-3 FA.

## Results

### Comparative effects of dietary TAG-DHA, PC-DHA, and LPC-DHA on brain DHA and BDNF in rats

**Figure 1:**
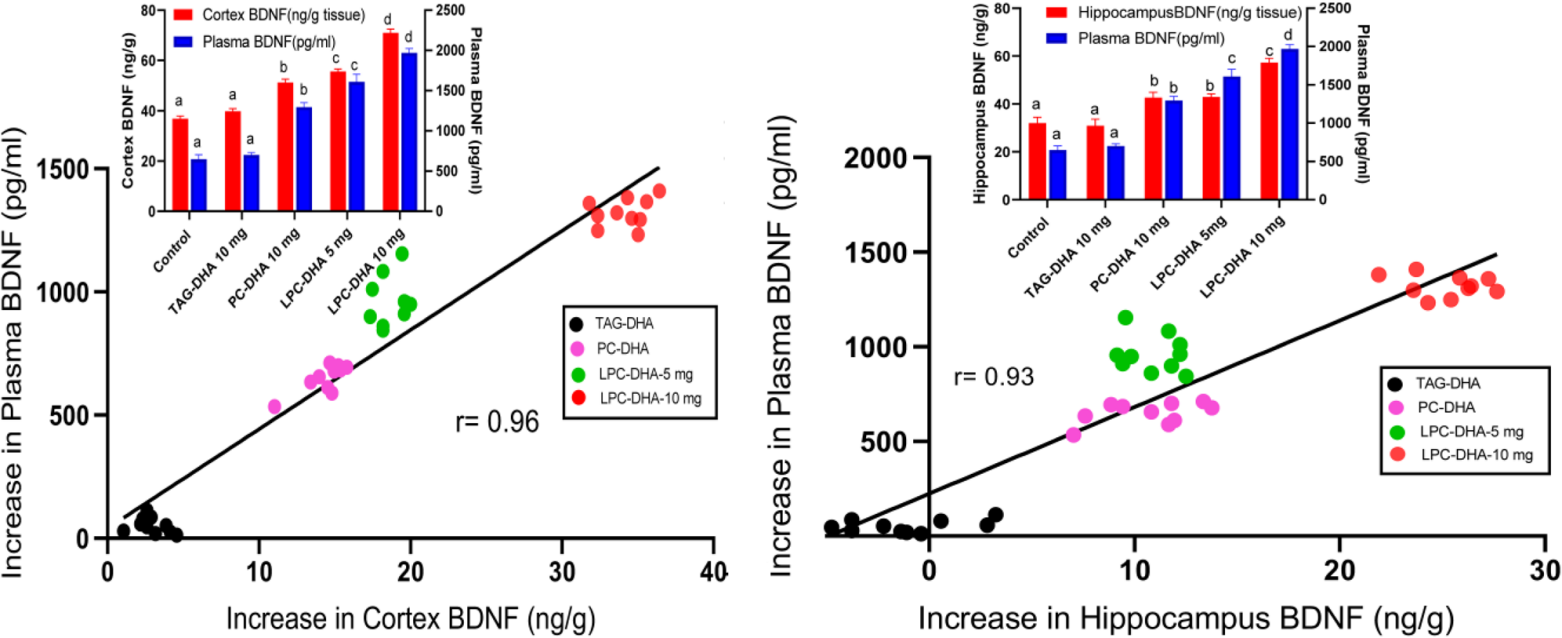
Correlation of plasma BDNF levels with brain BDNF in rats. Two month old rats were gavaged daily with the indicated DHA-compounds (40 mg DHA/kg body weight) for one month, and the BDNF levels in plasma and brain regions were determined by ELISA. Two doses of LPC-DHA (5 mg and 10 mg) equivalent to 20 mg DHA and 40 mg DHA/kg body weight respectively were used. The insets show the absolute values (mean ± SD, n = 10 rats/group) of BDNF in the control (untreated) and DHA-treated groups. Bars of the same color without common superscripts are significantly different from each other (one-way ANOVA, with Tukey multiple comparison correction). The increase in BDNF by DHA treatment was calculated by subtracting the average of the control values from the individual samples of the treated groups. Pearson correlation was calculated between the increase in plasma BDNF vs the increase in cortex or hippocampus (Graphpad, Prism 8.0).

**Figure 2:**
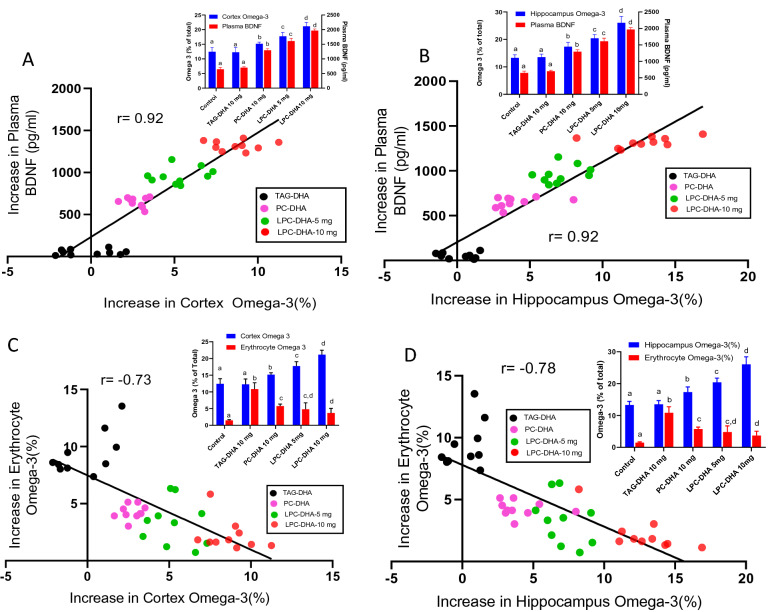
Correlation of omega-3 FA levels of brain with plasma BDNF and with erythrocyte omega-3 index in rats. The increase in cortex and hippocampus omega-3 FA (DHA + EPA) by the dietary treatment with various DHA carriers is plotted against the increase in plasma BDNF (**A** and **B** respectively) or against the erythrocyte omega-3 index (**C** and **D** respectively). The increase in brain omega-3 index was positively correlated with plasma BDNF, but negatively correlated with the increase in the erythrocyte index. The insets show the absolute values (mean ± SD, n = 10 rats/group), of BDNF and omega-3 FA (EPA + DHA), including those of the controls. Bars of same color without common superscripts are significantly different from each other by one-way ANOVA, with Tukey post-hoc correction.

Our previous studies in rats showed that the brain DHA levels can be altered over a wide range by feeding different molecular carriers of DHA^24,25^. Whereas triacylglycerol (TAG)-DHA had minimal effect on brain DHA, di-DHA PC (phosphatidylcholine) and LPC-DHA markedly and dose dependently increased the DHA in all regions of the brain^25^. Furthermore, brain BDNF levels were increased significantly in proportion to the increase in DHA^24^. Since there is a bidirectional transport of BDNF across the BBB^28^, we determined whether the increase in brain BDNF also results in an increase in plasma BDNF. As shown in Fig. 1, there was indeed a positive correlation between the increase in plasma BDNF and the increases in BDNF levels of cortex and hippocampus after treatment with various molecular carriers of dietary DHA. The absolute values of the BDNF (and the statistical significance determined by ANOVA) are shown in the insets. These results show that the changes in plasma BDNF levels reflect the changes in brain BDNF levels, as also reported by others^29^. Since the increase in brain BDNF is correlated with the increase in brain DHA^24^, we tested the hypothesis that plasma BDNF may be a valid biomarker for the changes in the brain DHA content. As shown in Fig. 2A, B, the increase in plasma BDNF correlated positively with the increase in DHA in both cortex and hippocampus over a wide range of values. The insets show absolute percentages of brain omega-3 FA (EPA + DHA) and the concentrations of plasma BDNF under various dietary conditions. We have also determined the correlation between the increases in brain DHA levels and erythrocyte omega-3 index, the most commonly used biomarker for measuring the incorporation of dietary omega-3 FA into brain and other tissues^18,30^. As shown in Fig. 2C, D, the increases in erythrocyte omega-3 levels were actually negatively correlated with the increases in cortex or hippocampus omega-3 levels. This is due to the fact that TAG-DHA significantly increased the erythrocyte omega-3 FA without appreciably increasing the brain omega-3 FA. On the other hand, PC-DHA and LPC-DHA which increased the brain omega-3 FA, had only modest effect on erythrocytes. These results therefore show that the erythrocyte omega-3 index is not a suitable marker for the changes in brain omega-3 FA altered by dietary lipids.

**Figure 3:**
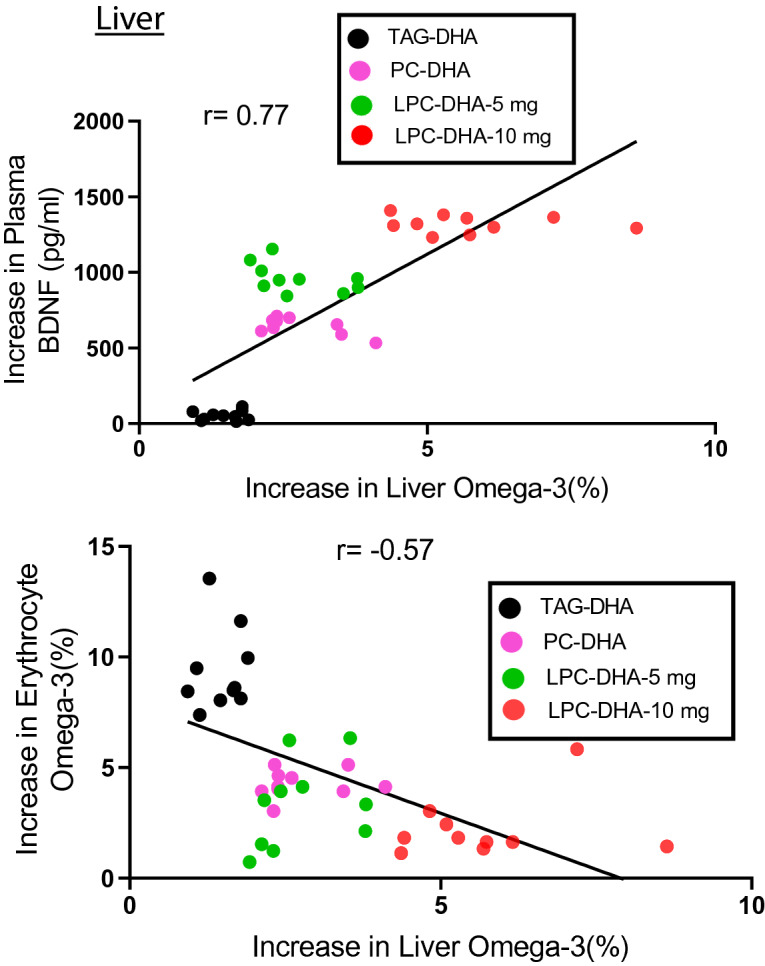
Correlation of liver omega-3 FA enrichment with plasma BDNF and erythrocyte omega-3 index in rats. The increase in omega-3 FA levels in the liver correlated positively with the increase in plasma BDNF (top), but correlated negatively with the increase in erythrocyte omega-3 index (bottom).

**Figure 4:**
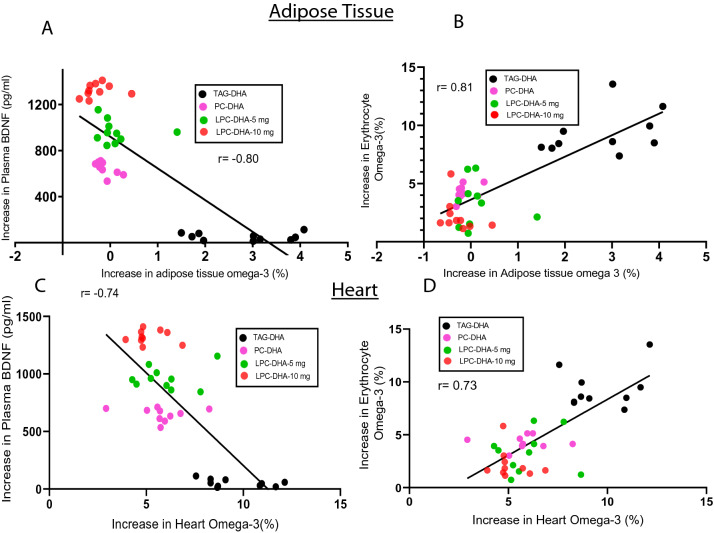
Correlation of brain omega-3 changes in adipose tissue (top) and heart (bottom) with changes in plasma BDNF or erythrocyte omega-3 index in rats. The increase in omega-3 index of peri-gonadal adipose tissue (**A**) and of heart (**C**) correlated negatively with the increase in plasma BDNF, whereas the they were correlated positively with the increase in erythrocyte omega-3 index (**B**, **D**).

It should be pointed out that the negative correlation observed above does not mean a reciprocal relationship between erythrocyte omega-3 and brain omega-3. Instead, this is due to the divergent mechanisms of uptake by the two tissues. Brain acquires its DHA through the Mfsd2a transporter pathway which prefers LPC-DHA over other forms of DHA^23^, whereas the DHA uptake by the erythrocytes is most likely through the exchange with plasma lipids. Therefore, it is possible that the erythrocyte index may not reflect the brain index, but could reflect the uptake of DHA by other peripheral tissue that acquire DHA through non-Mfsd2a pathways, including uptake of free FA through diffusion, and the receptor-mediated uptake of lipoproteins. To investigate this, we determined the correlation of changes in erythrocyte omega-3 index with changes in this index of other tissues. In addition, we determined the correlation of the omega-3 index of these tissues with plasma BDNF. As shown in Fig. 3, the liver omega-3 index was negatively correlated with the erythrocyte index, but positively correlated with the increase in plasma BDNF, similar to the brain. In contrast, the changes in erythrocyte index were positively correlated with the changes in heart (Fig. 4A) and adipose tissue (Fig. 4C). In both these tissues, TAG-DHA was more efficient than LPC-DHA or PC-DHA in increasing the omega-3 FA^25^. The plasma BDNF changes, on the other hand, were negatively correlated with the changes in omega-3 index of adipose tissue (Fig. 4B) as well as heart (Fig. 4D). In conclusion, these results show that the erythrocyte index, which has been widely used as a surrogate for the tissue incorporation of dietary omega-3 FA, reflects only selected tissues such as adipose tissue and the heart, but not the brain or liver.

### Studies in normal mice; effect of dietary free (unesterified) DHA versus LPC-DHA

**Figure 5:**
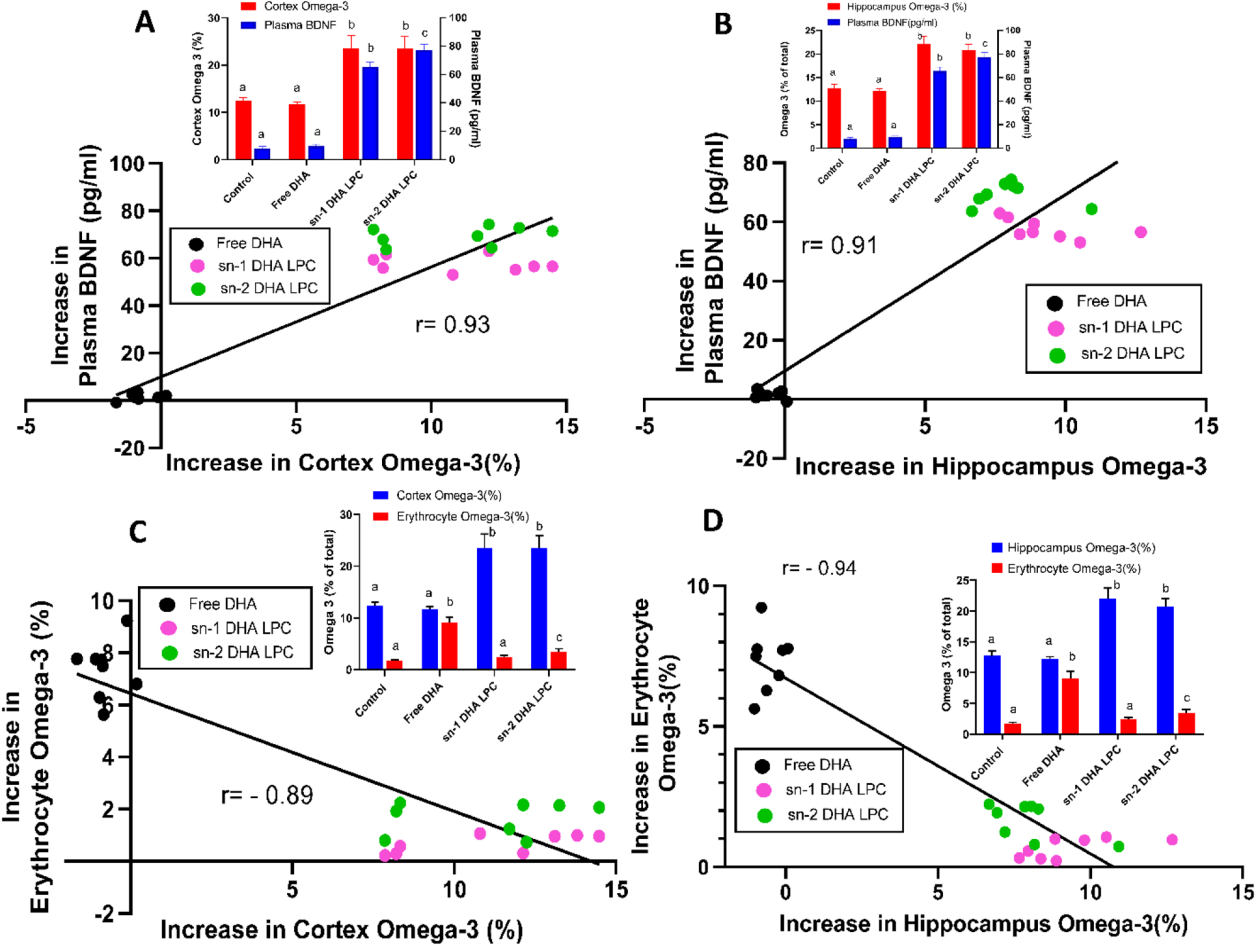
Correlation of brain omega-3 index with plasma BDNF and erythrocyte omega-3 index in normal mice. Normal male mice were gavaged with 40 mg DHA/kg body weight in the form of free DHA, sn-1 acyl LPC-DHA, or sn-2 acyl LPC-DHA for 30 days. The omega-3 FA content (DHA + EPA) of the brain regions and erythrocytes was measured by GC/MS, and the plasma BDNF levels were measured by ELISA. The insets show the absolute values (mean ± SD, n = 8 mice/group) for the % of omega-3 fatty acids (DHA + EPA) and the plasma BDNF values (pg/ml) in all groups, including controls (no treatment). In the inserts, bars of same color without common superscripts are significantly different from each other by one-way ANOVA, with Tukey post-hoc correction. The increases in omega-3 index and plasma BDNF levels above the control values are plotted. The increases in omega-3 FA of cortex as well as hippocampus correlated positively with the increases in plasma BDNF (**A**, **B**), whereas they correlated negatively with the omega-3 index of the erythrocytes (**C**, **D**).

Although some previous studies suggested that BDNF is absent in mouse plasma^29,31^, more recent studies showed the presence of measurable amounts^32,33^. We previously showed that while dietary free DHA did not appreciably increase brain DHA, LPC-DHA (both sn-1 acyl and sn-2 acyl isomers) markedly increased brain DHA, as well as BDNF, and improved brain function in normal male mice^24^. We now determined whether the increase in brain DHA by LPC-DHA resulted in an increase of plasma BDNF in mouse plasma also. As shown in Fig. 5A, B (insets), free DHA did not increase plasma BDNF levels compared to controls, whereas both isomers of LPC-DHA significantly increased it in cortex as well as hippocampus. There was a positive correlation between the increase in plasma BDNF and the increase in brain BDNF (Supplementary Figs. 1 and 2 online). The increase in plasma BDNF above the control value correlated positively with the increase in omega-3 index in both the brain regions. In contrast, the increase in erythrocyte index was negatively correlated with the increases in cortex and hippocampus (Fig. 5C, D). These results are similar to those obtained in rats, and thus show that plasma BDNF is a valid marker for changes in brain omega-3 FA levels not only in rats but also in mice.

### Effect of dietary LPC-EPA in mice

**Figure 6:**
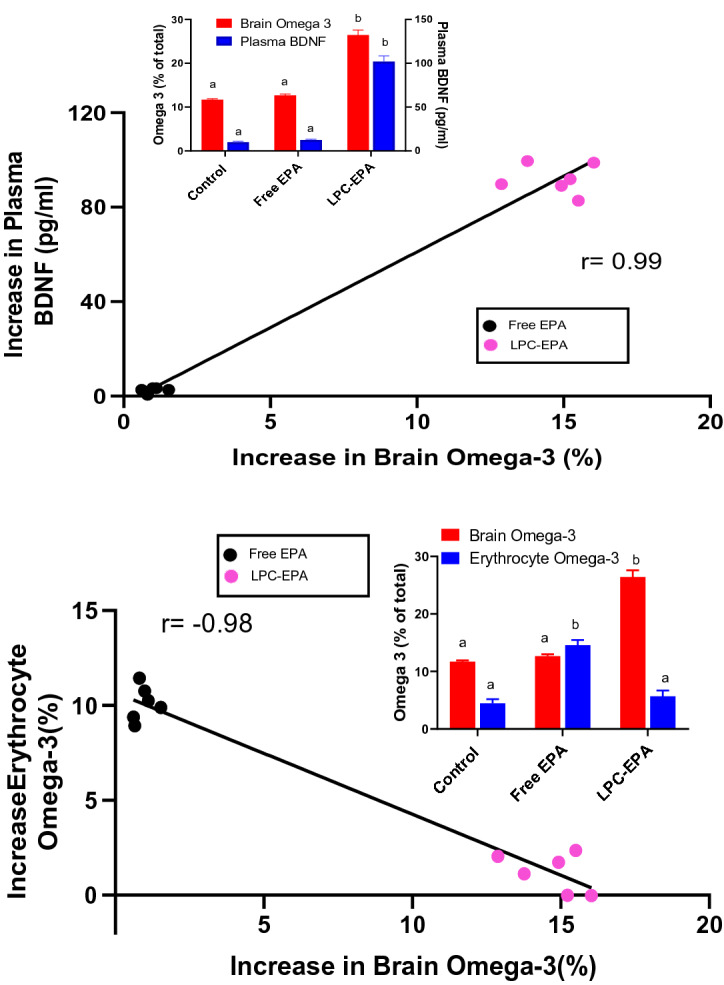
Correlation of brain omega-3 index with plasma BDNF or erythrocyte omega-3 index in mice fed EPA. Normal male mice were gavaged with 40 mg EPA/kg body weight in the form of either free (unesterified) EPA or LPC-EPA for 15 days, and the plasma BDNF as well as omega-3 indexes were measured. The increases in omega-3 index of the brain (over the averages of control values) are plotted against the increases in plasma BDNF (top) or erythrocyte omega-3 index (bottom). The absolute values of omega indexes (EPA + DHA) and plasma BDNF (pg/ml) for all groups including the control are shown in the insets (mean ± SD, n = 6 mice/group). In the inserts, bars of same color without common superscripts are significantly different from each other by one-way ANOVA.

Whereas previous studies reported that brain EPA levels cannot be increased through diet^34,35^, we have demonstrated that feeding LPC-EPA to normal mice not only increases brain EPA levels by several fold, but also increases brain DHA by about 100% in normal mice^36^. We determined whether the increase in plasma BDNF can be used as a biomarker for the increase in brain EPA and DHA after feeding free and LPC-EPA. As shown in Fig. 6, the increase in plasma BDNF above the average of control values positively correlated with the increase in the brain omega-3 index. However, the increase in erythrocyte omega-3 index was negatively correlated with that of brain because free EPA increased the omega-3 content of erythrocytes but not the brain, similar to the effects of TAG-DHA. The insets show the absolute values for all groups, including the controls. The increase in plasma BDNF also correlated positively with its increase in the brain (Supplementary Fig. 3 online). These results show that the effect of feeding LPC-EPA on plasma BDNF are similar to those of feeding LPC-DHA.

### Studies with lipase treated krill oil and fish oil

**Figure 7:**
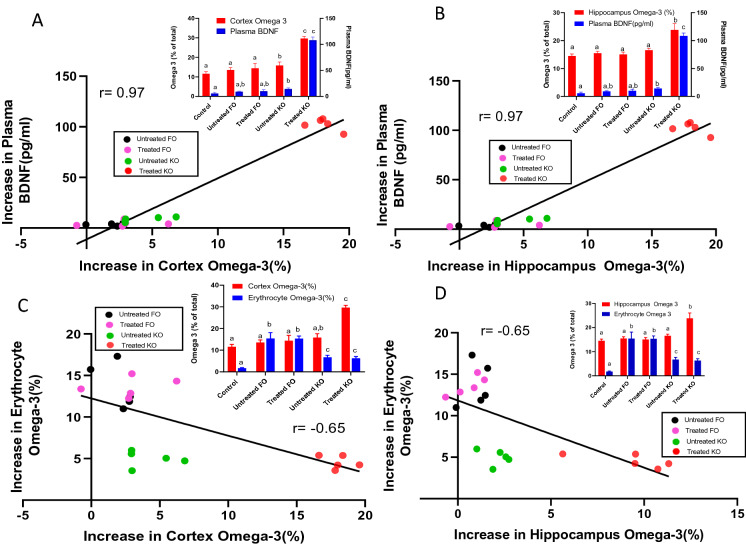
Correlation of brain omega-3 index with plasma BDNF and erythrocyte omega-3 index in mice fed krill oil or fish oil. Natural or lipase-treated fish oil and krill oil were incorporated into AIN-93G diet to provide 2.64 g of EPA + DHA per kg diet. These diets were fed to normal male mice for 30 days, and the tissue FA composition as well as plasma BDNF contents were measured. The top 2 panels (**A**, **B**) show the correlation of the increase in omega-3 indexes of cortex and hippocampus with the increase in plasma BDNF levels, whereas the bottom 2 panels (**C**, **D**) show the correlation of the increases in cortex and hippocampus omega-3 indexes with that of erythrocytes. The insets show the absolute values (mean ± SD, n = 5 mice/group) of omega-3 indexes (% of EPA + DHA) and the plasma BDNF levels (pg/ml) for all groups including the controls (which were fed unsupplemented AIN-93G diet). Bars of same color with different superscripts are significantly different from each other by one-way ANOVA.

We recently showed that the brain omega-3 index can be significantly increased by feeding krill oil which has been pre-treated with a lipase (thus generating LPC-EPA and LPC-DHA), but not by similarly treated fish oil, which cannot generate LPC^37^. Since many of the clinical studies are carried out with fish oil or krill oil, we determined whether the plasma BDNF can be used as surrogate for brain omega-3 index in the mice treated with the lipase-modified and unmodified krill oil and fish oil. As shown in Fig. 7A, B (insets), only the lipase-treated krill oil significantly increased the omega-3 FA content in both cortex and hippocampus. The increase in plasma BDNF correlated with its increase in cortex and hippocampus (Supplementary Figs. 4 and 5 online). Furthermore, the increase in plasma BDNF above the control value positively correlated with the increases in the omega-3 indexes of cortex and hippocampus. On the other hand, the increase in erythrocyte index was negatively correlated with the increases in the indexes in cortex and hippocampus (Fig. 7C, D). These results are similar to the results obtained with pure LPC-DHA or LPC-EPA in the mice.

## Discussion

Although there are several nutritional supplements of omega-3 FA in the market claiming to improve brain function and to protect against neurological diseases, controlled clinical trials supporting these claims are lacking. While some studies did report positive results^9–11^ many other studies reported negative results in improving brain function and memory^12–14^. An impediment for testing the effectiveness of the various supplements in humans is the lack of a reliable biomarker for the enrichment of brain omega-3 FA in response to them. Although the erythrocyte omega-3 index has been effectively used to evaluate the cardiovascular benefits of the omega-3 supplements^38^, the utility of this index in determining the effectiveness of these supplements for brain enrichment has not been demonstrated. In fact, the study by Fenton et al.^17^, which showed positive correlation of the erythrocyte omega-3 index with most other tissues excluded brain and liver, the two most important tissues relevant to the omega-3 FA function in the brain. Previous studies by Berliner et al.^21^ showed no significant correlation between the DHA concentration of erythrocyte membranes and that of brain membranes in miniature swine fed menhaden oil. Similarly, Tu et al.^22^ reported that after feeding α-linolenic acid to weanling rats, the erythrocyte omega-3 index correlated positively with that of most other tissues, but not the brain. Some epidemiologic studies also showed no correlation between erythrocyte index and depression^39^ or white matter hyperintensity^40^. Many experimental studies in animals, on the other hand, have reported a positive correlation between erythrocyte omega-3 index and that of the brain, but the range of brain DHA values achieved in these studies was narrow, since TAG-omega-3, which does not efficiently enrich brain omega-3, was used for feeding^19,22,41^. Interestingly we also found a positive correlation between the increase in erythrocyte and brain indexes if we plot only the values of the rats fed TAG-DHA (Supplementary Fig. 6 online). However, this correlation turned negative when the effects of PC-DHA and LPC-DHA are included, since the latter induce a much greater increase in brain DHA without a concomitant effect on erythrocytes (Fig. 2C, D). Therefore, the negative correlation does not mean that there is a reciprocal relationship between the erythrocytes and the brain, but instead is indicative of the divergent incorporation profiles of TAG-DHA and LPC-DHA. Whereas DHA from dietary TAG is incorporated significantly into erythrocytes, it is inefficient in enriching brain DHA. In contrast, LPC-DHA efficiently increased brain DHA by up to 100%, without significantly altering erythrocyte levels.

The current study makes a strong case for the plasma BDNF as a reliable non-invasive biomarker for the increase in brain omega-3 FA levels in response to treatments in patients. BDNF is an important neurotrophin with a role in neurogenesis, neuronal survival, learning and memory, as well as in regulation of body weight and energy homeostasis^42^. Plasma levels of BDNF are significantly decreased in patients with psychiatric disorders^43^, and are increased after treatment with anti-depressants^44^, as well as high doses of omega-3 FA^45^. Plasma BDNF is also significantly increased after vigorous exercise^46^, which is further enhanced by feeding DHA^27^. Importantly, it has been shown that there is a bidirectional transfer of BDNF between the brain and the plasma^27,28^, and that up to 80% of BDNF in the plasma may be derived from the brain^46^. There is convincing evidence that DHA increases the expression of BDNF in the brain possibly through the activation of Akt^27^ or GPR40^47^. Many of the beneficial effects of DHA may be through the expression of BDNF. Therefore, there is a physiological basis for using the plasma BDNF as a functional surrogate for brain omega-3 FA levels, unlike the erythrocyte omega-3 FA levels, which are metabolically unrelated to the brain levels.

In addition to the brain omega-3 FA status, the plasma BDNF may be a reliable marker for the omega-3 FA level of the liver, which does not correlate with the erythrocyte omega-3 index. There is ample evidence from experimental studies that high dietary omega-3 FA diets are beneficial in the treatment of fatty liver^48^, but mixed results were obtained in the clinical trials. Measuring plasma BDNF, which correlates positively with the hepatic enrichment of omega-3 FA would be helpful in determining the effectiveness of the treatments with nutritional supplements. It may be pointed out that the BDNF concentration of serum is much higher than the plasma, since large amounts of BDNF are released during the activation of platelets^49^. Therefore, it is important to measure the BDNF levels in the plasma, not in the serum, for more accurate reflection of the brain DHA levels. Another important consideration is that since BDNF expression and its plasma levels are also increased by vigorous exercise^46^ and anti-depressant treatments^44^, such factors should be controlled for, if present, in order to specifically measure the effects of DHA. For example, any exercise regimen and anti-depressant therapy should be continued as usual during omega-3 FA treatment, and plasma BDNF should be measured before and after the treatment period.

## Materials and methods

### Animals and dietary treatments

Most of the analyses were carried out on samples obtained from our studies published previously^24,25,36,37^. All animal protocols were approved by the UIC institutional animal care committee, and all methods were carried out in accordance with the relevant guidelines and regulations. Male Sprague–Dawley rats (8 week old) were purchased from Harlan laboratories (Indianapolis, IN). Male c57BL/6 mice (2–4 months old) were purchased from Jackson Laboratories (Bar Harbor. Maine).

In study 1, the rats were gavaged daily with 10 mg of DHA in the form of TAG-DHA, di-DHA PC, or 5 and 10 mg of DHA in the form of LPC-DHA for 30 days^25^. In study 2, male mice (4 month old) were gavaged daily with 40 mg DHA/kg body weight in the form of free DHA, sn-1 acyl LPC-DHA, or sn-2 acyl LPC-DHA for 30 days as described previously^24^. In study 3, male mice (2 month old) were gavaged daily with 40 mg EPA/kg body weight in the form of free EPA or LPC-EPA for 15 days, as described previously^36^. In study 4, male mice (2 month old) were fed diets enriched with natural or lipase-treated fish oil or natural or lipase-treated krill oil for 30 days. The FA composition of most of the tissues, determined by GC/MS, has been presented in our previous studies^24,25,36,37^. In addition, we analyzed the FA composition of the erythrocytes in all animals by GC/MS for this study. The values of EPA and DHA (percentage of total) were combined to give the omega-3 index of the tissues.

### Analytical procedures

The FA analysis of tissues was carried out by GC/MS as described previously^24^. BDNF in plasma and brain regions was assayed by ELISA, using Promega Emax Immunoassay system kit (Promega Inc., Madison, WI, USA), according to the manufacturer’s protocol. Rat brain regions (Cortex and hippocampus) were homogenized in the lysis buffer, and the homogenates were centrifuged at 10,000×*g*, for 20 min. The supernatants were collected and used for the quantification of BDNF levels.

### Statistics and correlations

The significance of differences between treatment groups was determined by one-way ANOVA, with Tukey post hoc multiple comparison corrections. For each study, the average of control values (untreated group) was first calculated. This average was then subtracted from individual values of the treatment groups to calculate the increases in omega-3 FA of tissues or plasma BDNF due to the treatment. The increases in omega-3 FA in the brain and other tissues were plotted against the increases in plasma BDNF or erythrocyte omega-3 of each animal to determine the Pearson correlation coefficients (Graphpad Prism 8.0).

## Supplementary information

Supplementary file1 (DOCX 1058 kb)
