# Supplementary material for: Plasma BDNF is a more reliable biomarker than erythrocyte omega-3 index for the omega-3 fatty acid enrichment of brain
Source: Sci Rep. 2020 Jul 2;10:10809. doi: 10.1038/s41598-020-67868-9 (PMC7331585; doi:10.1038/s41598-020-67868-9)

Supplementary Information

**Plasma BDNF is a more reliable biomarker than erythrocyte omega-3 index for the omega-3 fatty acid enrichment of brain**

Dhavamani Sugasini^1^, Poorna C.R. Yalagala^1^, and Papasani V. Subbaiah^1,2^ *

^1^Division of Endocrinology and Metabolism, Department of Medicine,

University of Illinois at Chicago, IL 60612,

and ^2^Jesse Brown VA Medical Center, Chicago, IL 60612

U.S.A

*****Address correspondence to**:**

P.V.Subbaiah, Ph.D.

Department of Medicine, University of Illinois at Chicago, Chicago, IL 60612

Email: [psubbaia@uic.edu](mailto:psubbaia@uic.edu)

Phone: 312-996-8212

**Supplementary Figure S1**

**Correlation of increase in plasma BDNF with the increase in cortex BDNF in mice**:

Normal mice were gavaged daily with 80 µl corn oil alone (control), or corn oil containing 1 mg DHA in the form of free (unesterified) DHA, sn-1 DHA LPC, or sn-2 DHA LPC for 30 days, and the BDNF values were determined by ELISA in the plasma and cortex (n=8 for all groups). The increase in BDNF values was calculated by subtracting the average of control values from the DHA-treated sample values. Pearson correlations were calculated by Graphpad Prism 8.0.

**
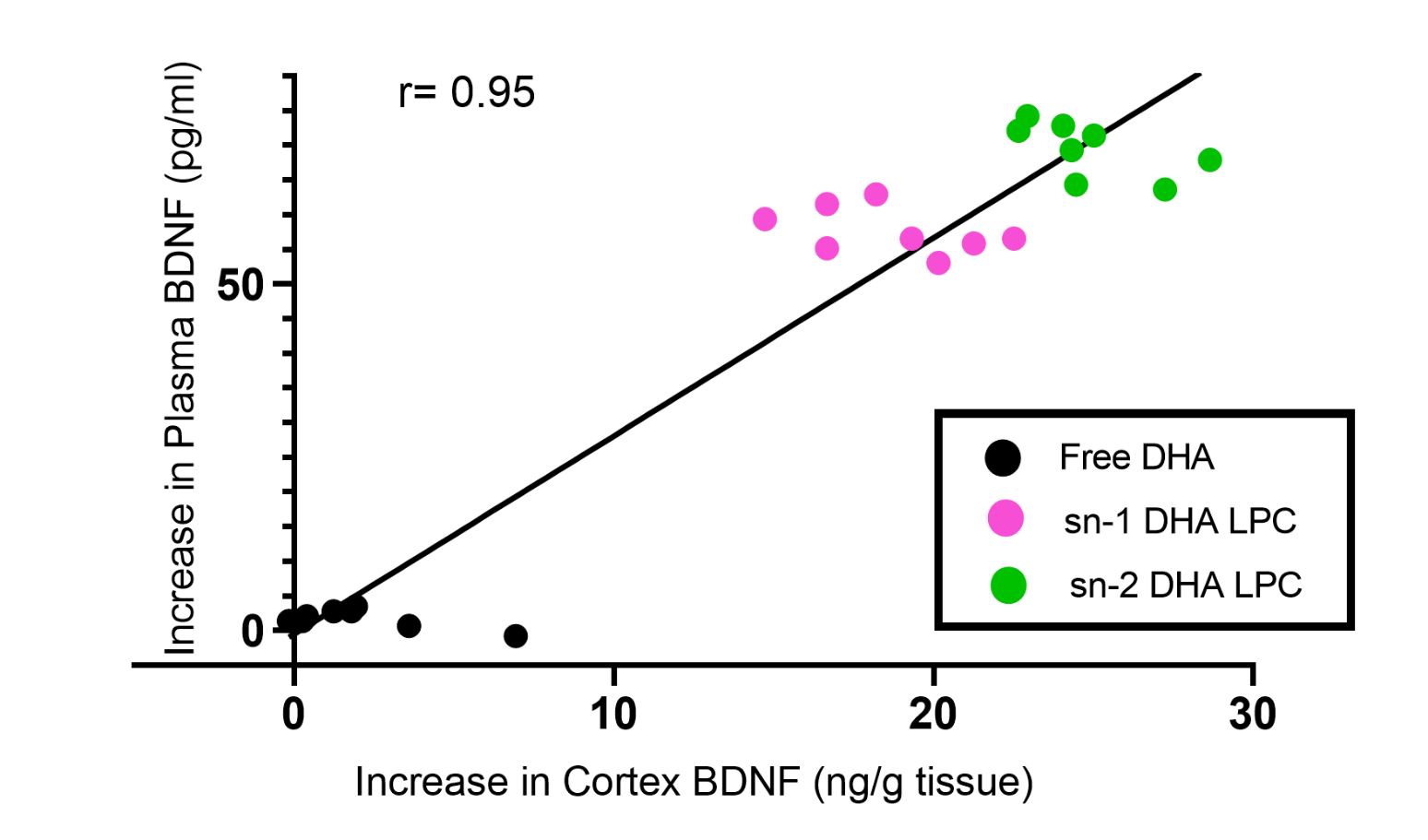
**

**Supplementary Figure S2**

**Correlation of increase in plasma BDNF with the increase in hippocampus BDNF in mice**:

Normal mice were gavaged daily with 80 µl corn oil alone (control), or corn oil containing 1 mg DHA in the form of free (unesterified) DHA, sn-1 DHA LPC, or sn-2 DHA LPC for 30 days, and the BDNF values were determined in the plasma and hippocampus. The increase in BDNF values was calculated by subtracting the average of the control values from the DHA-treated sample values. Pearson correlations were calculated by Graphpad Prism 8.0.

**
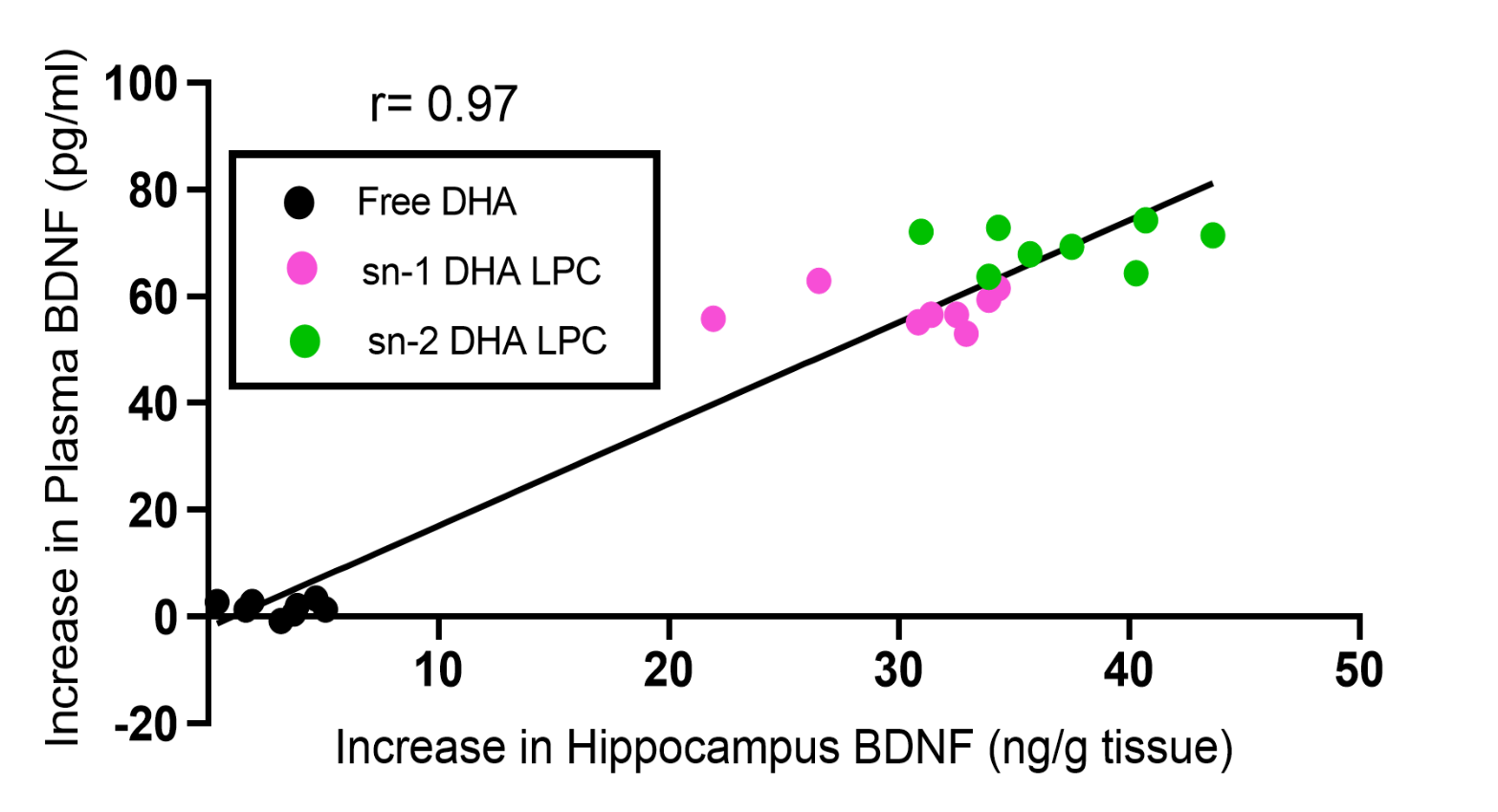
**

**Supplementary Figure S3**

**Correlation of increase in plasma BDNF with the increase in brain BDNF in mice treated with EPA**. Normal mice were gavaged daily with 80 µl of corn oil alone (control) or corn oil containing 1 mg EPA in the form of free (unesterified) EPA or LPC-EPA for 15 days (n=6 per group). BDNF was estimated by ELISA in the whole brain and plasma. The values shown are increases in BDNF over the average of control values. Pearson correlation was calculated in Graphpad Prism 8.0.


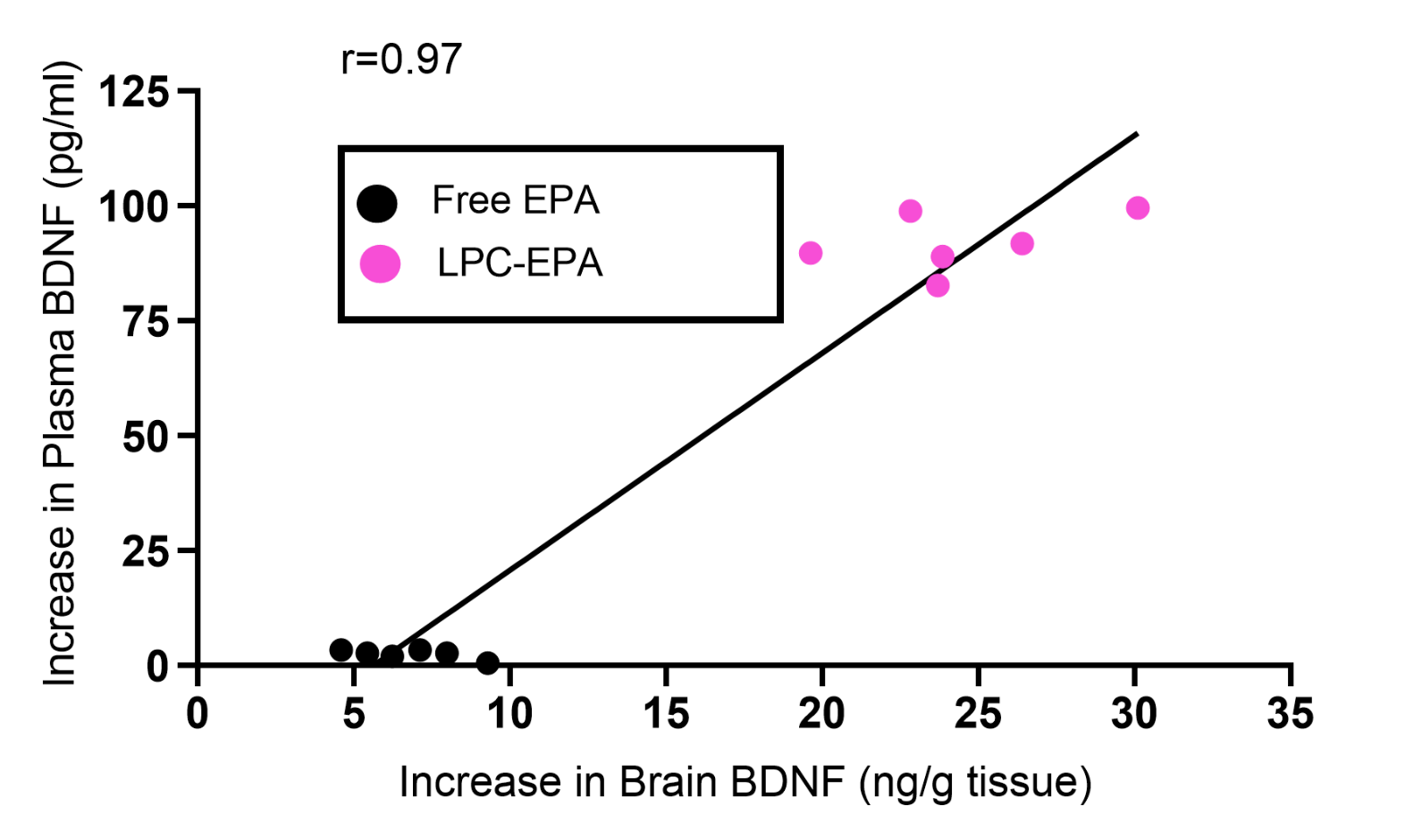


**Supplementary Figure S4**

**Correlation of increase in plasma BDNF with increase in cortex BDNF in mice fed fish oil or krill oil.** Mice were fed AIN-93G diet enriched with natural (untreated) fish oil, untreated krill oil, lipase-treated fish oil or lipase-treated krill oil for 30 days (total EPA+DHA, 2.64 g/kg diet). BDNF was determined in plasma and cortex by ELISA (n=5 per group). The increase in BDNF in individual mice was calculated by subtracting the average value of the control group, which were fed unsupplemented AIN-93 diet. Pearson correlation was calculated by Graphpad Prism 8.0.


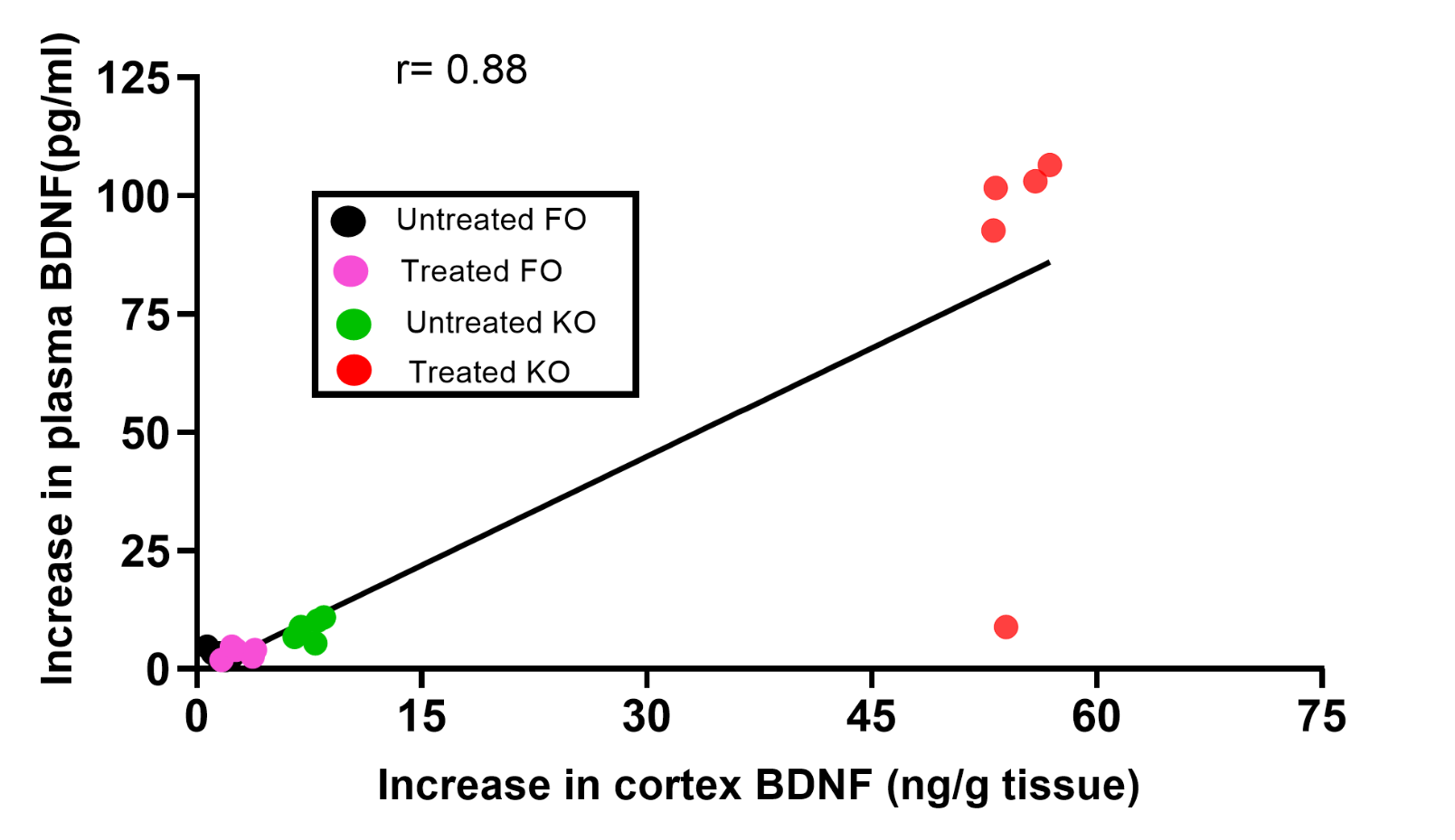


**Supplementary Figure S5**

**Correlation of increase in plasma BDNF with increase in hippocampus BDNF in mice fed fish oil or krill oil.** Mice were fed AIN-93G diet enriched with natural (untreated) fish oil, untreated krill oil, lipase-treated fish oil, or lipase-treated krill oil for 30 days (total EPA+DHA, 2.64 g/kg diet). BDNF was determined in plasma and hippocampus by ELISA (n=5 per group). The increase in BDNF was calculated by subtracting the average value of the control group, which were fed unsupplemented AIN-93 diet. Pearson correlation was calculated by Graphpad Prism 8.0.

**
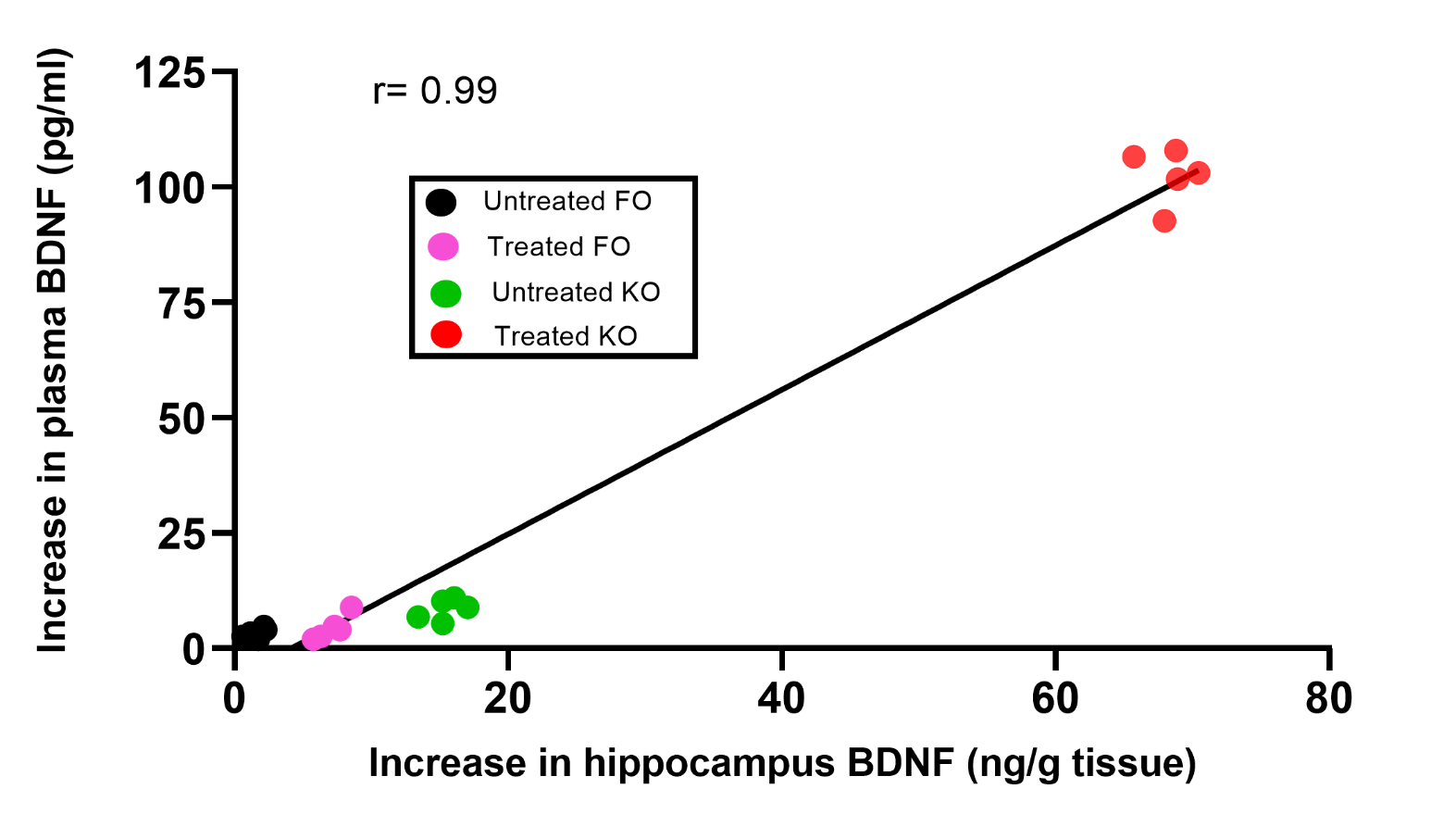
**

**Supplementary Figure S6**

**Correlation between the increase in brain BDNF and the increase in erythrocyte omega 3 index in rats fed TAG-DHA.**

A positive correlation is obtained if the values from TAG-DHA fed rats only are plotted. However, if the values from PC-DHA and LPC-DHA fed animals are also included, the relationship is negative, as shown in Figs. 2C and 2D in the main text.


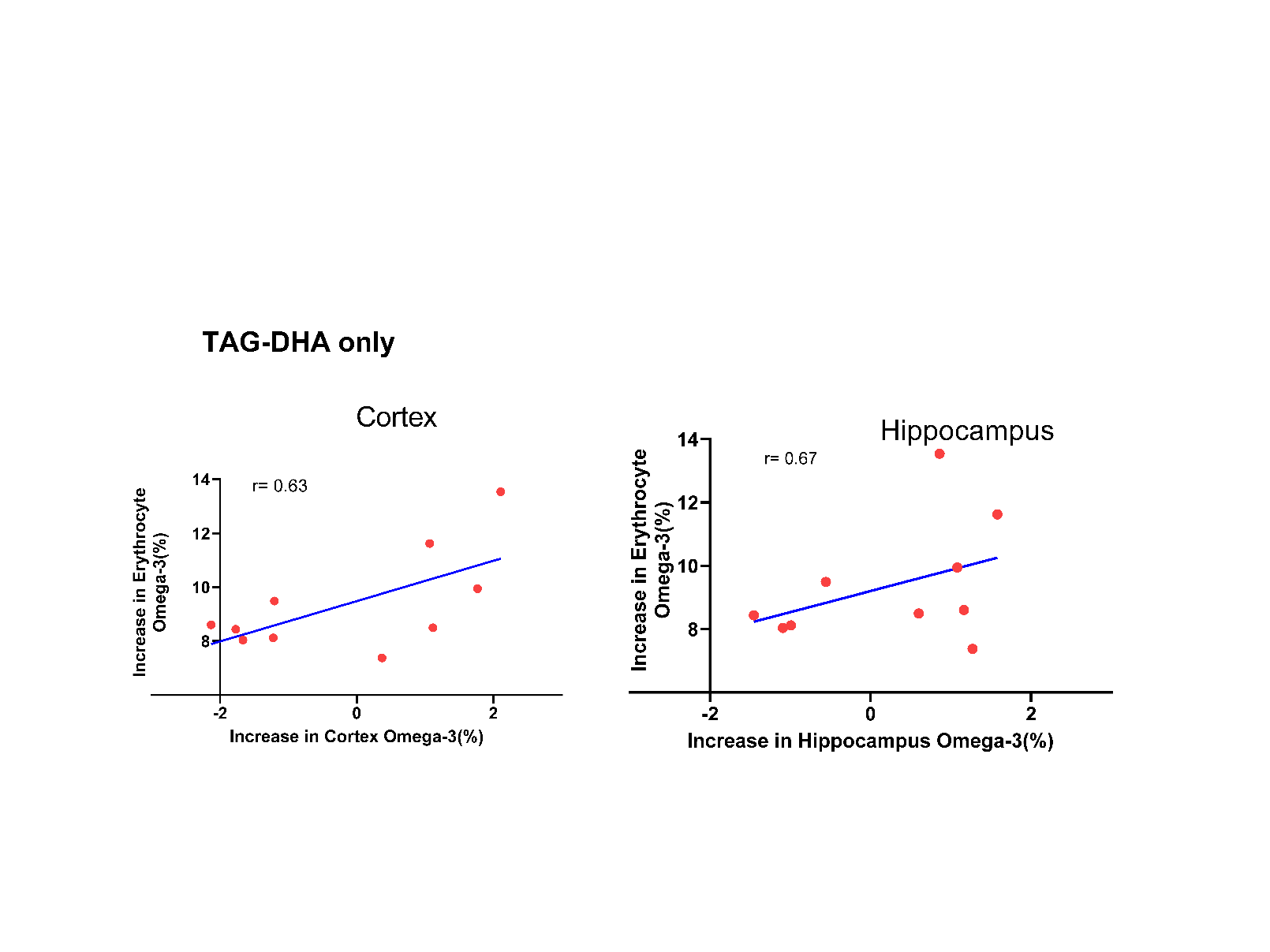

Supplement: Supplementary file 1 — Supplementary file1 (DOCX 1058 kb) [file 41598_2020_67868_MOESM1_ESM.docx]
